# Supplementary material for: Performance and Biomechanics in the Flight Period of Ski Jumping: Influence of Ski Attitude
Source: Biology (Basel). 2022 Apr 27;11(5):671. doi: 10.3390/biology11050671 (PMC9138498; doi:10.3390/biology11050671)
Supplement: Supplementary file 1 [file biology-11-00671-s001.zip › biology-1688883-supplementary.pdf]

biology

MDPI

Article

Performance and Biomechanics in the Flight Period of Ski Jumping: Influence of Ski Attitude

Lin Zhang [1], Xiong Li [2], Xin Wang [1,*], Long Chen [2], and Tianyu Zhao [2,*]

[1] Department of Kinesiology, Shenyang Sport University, Shenyang 110102, China
[2] Department of Mechanics, Northeastern University, Shenyang 110819, China
* Correspondence: wangxin@syty.edu.cn (X.W.); zhaotianyu@mail.neu.edu.cn (T.Z.)

Citation: Zhang, L.; Li, X.; Wang, X.; Chen, L.; Zhao, T. Performance and Biomechanics in the Flight Period of Ski Jumping: Influence of Ski Attitude. *Biology* **2022**, *11*, 671. https://doi.org/10.3390/biology11050671

Academic Editors: Filip Kukić, Jay Dawes, Robin Orr and Katie M. Heinrich

Received: 4 April 2022
Accepted: 24 April 2022
Published: 27 April 2022

Publisher's Note: MDPI stays neutral with regard to jurisdictional claims in published maps and institutional affiliations.

Copyright: © 2022 by the authors. Licensee MDPI, Basel, Switzerland. This article is an open access article distributed under the terms and conditions of the Creative Commons Attribution (CC BY) license (https://creativecommons.org/licenses/by/4.0/).

Supplementary Materials:

Table S1. The database for the aerodynamic forces and moments of the ski jumping ski.

α/°	β/°	γ/°	C_L	C_D	C_L / C_D	M_S	M_L	M_D
		0	3.84×10^{-4}	1.29×10^{-2}	2.98×10^{-2}	-4.43×10^{-4}	3.04×10^{-4}	-7.06×10^{-6}
		10	3.77×10^{-4}	1.29×10^{-2}	2.92×10^{-2}	-1.24×10^{-3}	8.96×10^{-5}	-7.06×10^{-6}
	0	20	3.59×10^{-4}	1.29×10^{-2}	2.78×10^{-2}	-1.23×10^{-3}	-1.27×10^{-4}	-7.06×10^{-6}
		30	3.29×10^{-4}	1.29×10^{-2}	2.55×10^{-2}	-1.19×10^{-3}	-3.40×10^{-4}	-7.06×10^{-6}
		40	2.9×10^{-4}	1.29×10^{-2}	2.25×10^{-2}	-1.11×10^{-3}	-5.41×10^{-4}	-7.06×10^{-6}
		0	8.48×10^{-3}	1.53×10^{-2}	0.554	-3.38×10^{-3}	-8.57×10^{-4}	-9.27×10^{-4}
		10	0.0361	1.72×10-2	2.12	-7.09×10^{-3}	-1.97×10^{-3}	1.56×10^{-4}
	10	20	0.0513	2.03×10-2	2.53	-1.11×10^{-2}	-3.89×10^{-3}	3.76×10^{-4}
		30	0.0550	2.32×10-2	2.37	-1.32×10^{-2}	-7.15×10^{-3}	4.97×10^{-4}
		40	0.0572	2.63×10-2	2.18	-1.36×10^{-2}	-1.02×10^{-2}	3.17×10^{-4}
		0	0.0205	1.99×10^{-2}	1.03	-7.85×10^{-3}	-2.10×10^{-3}	2.27×10^{-3}
		10	0.114	2.88×10^{-2}	3.96	-2.52×10^{-2}	-6.13×10^{-3}	5.85×10^{-3}
0	20	20	0.157	4.52×10^{-2}	3.47	-3.13×10^{-2}	-1.34×10^{-2}	6.40×10^{-3}
		30	0.187	6.63×10^{-2}	2.83	-3.84×10^{-2}	-2.29×10^{-2}	7.70×10^{-3}
		40	0.208	8.82×10^{-2}	2.36	-3.10×10^{-2}	-3.10×10^{-2}	6.53×10^{-3}
		0	0.0298	2.86×10^{-2}	1.04	-1.10×10^{-2}	-3.60×10^{-3}	5.49×10^{-3}
		10	0.208	5.12×10^{-2}	4.06	-4.35×10^{-2}	-1.12×10^{-2}	1.87×10^{-3}
	30	20	0.291	9.46×10^{-2}	3.08	-5.64×10^{-2}	-2.67×10^{-2}	2.27×10^{-3}
		30	0.422	1.62×10^{-2}	2.61	-7.75×10^{-2}	-2.42×10^{-2}	2.72×10^{-3}
		40	0.387	2.07×10^{-2}	1.87	-6.89×10^{-2}	-6.12×10^{-2}	2.45×10^{-3}
		0	0.0338	4.13×10^{-2}	0.819	-1.19×10^{-2}	-5.13×10^{-3}	8.97×10^{-3}
		10	0.303	8.51×10^{-2}	3.56	-5.27×10^{-2}	-1.55×10^{-2}	3.39×10^{-3}
	40	20	0.406	1.57×10^{-2}	2.59	-6.72×10^{-2}	-3.59×10^{-2}	4.08×10^{-3}
		30	0.497	2.52×10^{-2}	1.97	-7.72×10^{-2}	-5.93×10^{-2}	4.40×10^{-3}
		40	0.536	3.42×10^{-2}	1.57	-8.25×10^{-2}	-8.27×10^{-2}	4.48×10^{-3}
		0	0.0862	3.48×10^{-2}	2.48	-2.39×10^{-2}	7.95×10^{-4}	-1.95×10^{-3}
10	0	10	0.0886	3.50×10^{-2}	2.53	-2.32×10^{-2}	-3.25×10^{-3}	-2.56×10^{-3}
		20	0.0876	3.44×10^{-2}	2.55	-2.16×10^{-2}	-7.38×10^{-3}	-3.29×10^{-3}

		30	0.0816	3.28×10^{-2}	2.48	-1.80×10^{-2}	-1.06×10^{-2}	-3.90×10^{-3}
		40	0.0680	2.98×10^{-2}	2.27	-1.38×10^{-2}	-1.19×10^{-2}	-4.01×10^{-3}
	10	0	0.156	4.80×10^{-2}	3.25	-2.92×10^{-2}	4.89×10^{-4}	1.13×10^{-3}
		10	0.174	5.75×10^{-2}	3.02	-3.23×10^{-2}	-4.63×10^{-3}	3.62×10^{-4}
		20	0.166	6.28×10^{-2}	2.64	-3.37×10^{-2}	-1.04×10^{-2}	-4.34×10^{-4}
		30	0.136	6.06×10^{-2}	2.24	-3.45×10^{-2}	-1.84×10^{-2}	-1.36×10^{-3}
		40	0.111	5.93×10^{-2}	1.86	-2.97×10^{-2}	-2.36×10^{-2}	-2.67×10^{-3}
	20	0	0.248	7.40×10^{-2}	3.34	-5.02×10^{-2}	-3.74×10^{-3}	1.07×10^{-2}
		10	0.296	0.102	2.91	-5.82×10^{-2}	-1.19×10^{-2}	1.09×10^{-2}
		20	0.347	0.135	2.56	-5.93×10^{-2}	-2.04×10^{-2}	8.27×10^{-3}
		30	0.369	0.172	2.15	-5.67×10^{-2}	-3.10×10^{-2}	3.79×10^{-3}
		40	0.329	0.187	1.75	-4.54×10^{-2}	-3.84×10^{-2}	-2.28×10^{-3}
	30	0	0.328	0.100	3.28	-6.42×10^{-2}	-9.46×10^{-3}	2.55×10-2
		10	0.422	0.162	2.61	-7.75×10^{-2}	-2.42×10^{-2}	2.72×10-2
		20	0.480	0.223	2.15	-8.86×10^{-2}	-4.07×10^{-2}	2.84×10-2
		30	0.554	0.309	1.79	-8.97×10^{-2}	-5.85×10^{-2}	2.26×10-2
		40	0.554	0.396	1.39	-8.68×10^{-2}	-7.73×10-2	1.50×10-2
	40	0	0.382	0.125	3.06	-6.58×10-2	-1.32×10^{-2}	4.01×10^{-2}
		10	0.501	0.214	2.35	-8.27×10-2	-3.35×10^{-2}	4.54×10^{-2}
		20	0.587	0.313	1.88	-9.49×10-2	-5.56×10^{-2}	4.72×10^{-2}
		30	0.630	0.403	1.56	-0.102	-7.78×10^{-2}	4.59×10^{-2}
		40	0.613	0.530	1.16	-0.109	-0.113	4.26×10^{-2}
20	0	0	0.239	0.114	2.11	-5.92×10^{-2}	2.46×10^{-3}	-5.47×10^{-3}
		10	0.251	0.118	2.13	-6.10×10^{-2}	-8.33×10^{-3}	-9.32×10^{-3}
		20	0.308	0.140	2.20	-4.92×10^{-2}	-1.26×10^{-2}	-1.25×10^{-2}
		30	0.307	0.139	2.22	-4.33×10^{-2}	-2.07×10^{-2}	-1.75×10^{-2}
		40	0.246	0.115	2.14	3.91×10^{-2}	-2.81×10^{-2}	-1.82×10^{-2}
	10	0	0.432	0.186	2.33	-6.18×10^{-2}	4.37×10^{-5}	-7.21×10^{-4}
		10	0.340	0.164	2.07	-6.49×10^{-2}	-1.12×10^{-2}	-2.04×10^{-3}
		20	0.266	0.145	1.84	-6.52×10^{-2}	-2.26×10^{-2}	-4.18×10^{-3}
		30	0.240	0.144	1.67	-5.89×10^{-2}	-3.31×10^{-2}	-8.32×10^{-3}
		40	0.242	0.159	1.52	-5.32×10^{-2}	-4.14×10^{-2}	-1.34×10^{-2}
	20	0	0.515	0.220	2.34	-9.01×10^{-2}	-6.13×10^{-3}	1.75×10^{-2}
		10	0.581	0.289	2.01	-9.74×10^{-2}	-1.96×10^{-2}	1.34×10^{-2}
		20	0.569	0.330	1.72	-8.60×10^{-2}	-2.96×10^{-2}	4.22×10^{-3}
		30	0.382	0.263	1.45	-7.85×10^{-2}	-4.75×10^{-2}	-1.33×10^{-3}
		40	0.279	0.236	1.18	-6.85×10^{-2}	-5.89×10^{-2}	-6.73×10^{-3}
	30	0	0.561	0.255	2.20	-9.79×10^{-2}	-1.77×10^{-2}	3.44×10^{-2}
		10	0.634	0.338	1.88	-0.115	-3.60×10^{-2}	3.58×10^{-2}
		20	0.729	0.475	1.54	-0.121	-5.72×10^{-2}	2.76×10^{-2}
		30	0.715	0.569	1.26	-0.108	-7.35×10^{-2}	1.03×10^{-2}
		40	0.441	0.451	0.977	-9.33×10^{-2}	-8.61×10^{-2}	3.70×10^{-3}

	40	0	0.590	0.282	2.10	-9.76×10^{-2}	-2.85×10^{-2}	5.30×10^{-2}
		10	0.679	0.387	1.75	-0.114	-5.02×10^{-2}	5.50×10^{-2}
		20	0.703	0.492	1.43	-0.125	-7.69×10^{-2}	5.22×10^{-2}
		30	0.678	0.622	1.09	-0.132	-0.111	4.30×10^{-2}
		40	0.581	0.703	0.826	-0.114	-0.123	2.20×10^{-2}
30	0	0	0.414	0.272	1.52	-9.75×10^{-2}	6.05×10^{-3}	-9.49×10^{-3}
		10	0.437	0.286	1.52	-0.102	-9.12×10^{-3}	-1.82×10^{-2}
		20	0.604	0.386	1.56	-8.85×10^{-2}	-2.24×10^{-2}	-3.23×10^{-2}
		30	0.560	0.359	1.56	-9.48×10^{-2}	-4.02×10^{-2}	-4.27×10^{-2}
		40	0.441	0.288	1.53	-7.89×10^{-2}	-4.81×10^{-2}	-4.47×10^{-2}
	10	0	0.634	0.408	1.56	-9.99×10^{-2}	4.09×10^{-3}	1.92×10^{-3}
		10	0.445	0.311	1.43	-0.105	-1.84×10^{-2}	-4.71×10^{-3}
		20	0.406	0.305	1.33	-9.84×10^{-2}	-3.26×10^{-2}	-1.27×10^{-2}
		30	0.427	0.344	1.23	-9.44×10^{-2}	-4.82×10^{-2}	-2.40×10^{-2}
		40	0.499	0.431	1.16	-8.69×10^{-2}	-6.20×10^{-2}	-3.99×10^{-2}
	20	0	0.813	0.515	1.58	-0.134	-1.22×10^{-2}	2.18×10^{-2}
		10	0.794	0.571	1.40	-0.123	-2.87×10^{-2}	6.35×10^{-3}
		20	0.502	0.420	1.20	-0.118	-4.99×10^{-2}	2.43×10^{-4}
		30	0.408	0.395	1.03	-0.101	-6.17×10^{-2}	-1.09×10^{-2}
		40	0.360	0.414	0.870	-9.64×10^{-2}	-7.91×10^{-2}	-2.28×10^{-2}
	30	0	0.726	0.456	1.59	-0.136	-2.86×10^{-2}	4.56×10^{-2}
		10	0.766	0.585	1.31	-0.155	-5.72×10^{-2}	3.90×10^{-2}
		20	0.749	0.690	1.09	-0.138	-7.03×10^{-2}	1.71×10^{-2}
		30	0.506	0.571	0.887	-0.122	-8.81×10^{-2}	3.25×10^{-3}
		40	0.382	0.548	0.698	-0.102	-9.68×10^{-2}	-1.12×10^{-2}
	40	0	0.730	0.459	1.59	-0.126	-4.11×10^{-2}	6.49×10^{-2}
		10	0.752	0.588	1.28	-0.142	-7.03×10^{-2}	6.01×10^{-2}
		20	0.723	0.721	1.002	-0.148	-0.101	4.59×10^{-2}
		30	0.605	0.774	0.781	-0.134	-0.112	2.38×10^{-2}
		40	0.435	0.756	0.575	-0.114	-0.127	1.52×10^{-3}
40	0	0	0.556	0.509	1.09	-0.135	-8.70×10^{-2}	-1.25×10^{-2}
		10	0.579	0.531	1.09	-0.142	-6.99×10^{-3}	-2.80×10^{-2}
		20	0.734	0.664	1.10	-0.133	-2.06×10^{-2}	-4.66×10^{-2}
		30	0.653	0.592	1.10	-0.140	-4.74×10^{-2}	-6.81×10^{-2}
		40	0.495	0.454	1.09	-0.111	-5.67×10^{-2}	-7.09×10^{-2}
	10	0	0.619	0.564	1.10	-0.149	-3.78×10^{-3}	4.31×10^{-3}
		10	0.553	0.537	1.03	-0.137	-2.24×10^{-2}	-1.10×10^{-2}
		20	0.532	0.553	0.963	-0.138	-4.10×10^{-2}	-2.57×10^{-2}
		30	0.577	0.640	0.901	-0.131	-5.65×10^{-2}	-4.47×10^{-2}
		40	0.514	0.618	0.834	-0.131	-8.18×10^{-2}	-6.73×10^{-2}
	20	0	0.776	0.701	1.11	-0.169	-2.30×10^{-2}	2.59×10^{-2}
		10	0.628	0.642	0.979	-0.154	-4.23×10^{-2}	7.04×10^{-3}

	20	0.531	0.618	0.860	-0.138	-5.80×10^{-2}	-1.05×10^{-2}
	30	0.470	0.632	0.743	-0.133	-7.59×10^{-2}	-2.68×10^{-2}
	40	0.420	0.675	0.623	-0.126	-9.43×10^{-2}	-4.81×10^{-2}
30	0	0.770	0.688	1.12	-0.170	-4.61×10^{-2}	5.09×10^{-2}
	10	0.725	0.771	0.939	-0.167	-6.69×10^{-2}	2.99×10^{-2}
	20	0.588	0.758	0.776	-0.152	-8.41×10^{-2}	7.55×10^{-3}
	30	0.467	0.744	0.627	-0.134	-9.56×10^{-2}	-1.28×10^{-3}
	40	0.363	0.759	0.478	-0.125	-0.113	-3.30×10^{-2}
40	0	0.784	0.687	1.14	-0.148	-5.80×10^{-2}	6.81×10^{-2}
	10	0.7445	0.819	0.909	-0.156	-8.50×10^{-2}	5.20×10^{-2}
	20	0.639	0.896	0.714	-0.152	-0.108	2.91×10^{-2}
	30	0.488	0.9110	0.536	-0.139	-0.124	3.32×10^{-3}
	40	0.338	0.905	0.373	-0.122	-0.133	-1.97×10^{-2}
